# *OsSCYL2* is Involved in Regulating ABA Signaling-Mediated Seed Germination in Rice

**DOI:** 10.3390/plants13081088

**Published:** 2024-04-12

**Authors:** Minyan Xu, Wei Zhang, Yuhuan Jiao, Qing Yang, Meng Chen, Hu Cheng, Beijiu Cheng, Xin Zhang

**Affiliations:** National Engineering Laboratory of Crop Stress Resistance Breeding, School of Life Sciences, Anhui Agricultural University, Hefei 230036, China

**Keywords:** seed germination, ABA signaling, gene regulation, *OsSCYL2*, OsTOR

## Abstract

Seed germination represents a multifaceted biological process influenced by various intrinsic and extrinsic factors. In the present study, our investigation unveiled the regulatory role of *OsSCYL2*, a gene identified as a facilitator of seed germination in rice. Notably, the germination kinetics of *OsSCYL2*-overexpressing seeds surpassed those of their wild-type counterparts, indicating the potency of *OsSCYL2* in enhancing this developmental process. Moreover, qRT-PCR results showed that *OsSCYL2* was consistently expressed throughout the germination process in rice. Exogenous application of ABA on seeds and seedlings underscored the sensitivity of *OsSCYL2* to ABA during both seed germination initiation and post-germination growth phases. Transcriptomic profiling following *OsSCYL2* overexpression revealed profound alterations in metabolic pathways, MAPK signaling cascades, and phytohormone-mediated signal transduction pathways, with 15 genes related to the ABA pathways exhibiting significant expression changes. Complementary in vivo and in vitro assays unveiled the physical interaction between OsSCYL2 and TOR, thereby implicating OsSCYL2 in the negative modulation of ABA-responsive genes and its consequential impact on seed germination dynamics. This study elucidated novel insights into the function of Os*SCYL2* in regulating the germination process of rice seeds through the modulation of ABA signaling pathways, thereby enhancing the understanding of the functional significance of the SCYL protein family in plant physiological processes.

## 1. Introduction

Rice plays a crucial role in ensuring global food security due to its significance as a staple food. The rapid and uniform germination of seeds is indispensable for robust seedling growth [1]. This germination process is intricately influenced by both endogenous hormonal factors and environmental conditions. Among these hormones, abscisic acid (ABA) holds particular importance in plant physiology, regulating diverse processes such as seed dormancy and germination, growth modulation, flowering and fruiting promotion, and responses to external environmental stresses [2,3,4,5]. The cellular concentration of ABA is governed by a dynamic equilibrium between its biosynthesis and catabolism [6]. Expression levels of *OsNCEDs*, enzymes involved in ABA biosynthesis, exhibit a close association with endogenous ABA levels, with phenotypic variations in dormancy reduction or germination promotion often coinciding with the downregulation of all *OsNCEDs* except *OsNCED1* [7,8,9,10,11,12]. ABA catabolism predominantly relies on the hydroxylation activity of ABA 8′-hydroxylase (*ABA8ox*), and a decrease in the expression of the *OsABA8ox* gene has been observed to impede seed germination [13,14]. In addition to ABA metabolism, ABA signaling pathways also modulate seed germination. This pathway comprises ABA receptor PYR/PYL/RCAR proteins, protein phosphatase 2C (PP2C), and SNF1-related protein kinase 2 (SnRK2). Upon ABA presence, PYLs bind to ABA, initiating the signaling cascade, forming a complex with PP2Cs, thereby hindering PP2C-mediated dephosphorylation of SnRK2s [15,16]. Activated SnRK2s then phosphorylate transcription factors (TFs), triggering downstream target gene expression and associated plant responses, notably inhibiting seed germination [17,18,19].

Numerous TFs participate in ABA-mediated germination, encompassing *ABI3*, *AP2*, *bZIPs*, *WRKY*, and *NAC* TFs. *ABI3*, a prototypical B3 domain TF, positively modulates the ABA signaling pathway via its interaction with AREB/ABF/ABI5, which is crucial for embryonic growth during germination [19,20]. *OsAP2*-39 enhances *OsNCED1* expression, negatively influencing seed germination [21]. *ARAG1* (ABA-responsive AP2-like gene) and select *OsDREBs* contribute to rice seed germination processes [22]. *bZIPs* as AREB/ABF-type TFs, constitute the core downstream component and regulate a series of developmental events, including seed germination, photomorphogenesis, and response to stresses [23,24,25]. Among *bZIPs*, functioning as AREB/ABF-type TFs, *ABI5* (*OsbZIP10*), *ABF2* (*OsbZIP46*), and *OsbZIP75* promote ABA signaling while inhibiting seed germination [26,27,28]. Conversely, *OsbZIP09* facilitates seed germination by suppressing *OsLEAs* transcription and enhancing *OsLOX2* expression [29]. Additionally, *WRKY* and *NAC* TFs play regulatory roles in germination [10,30,31,32].

The target of rapamycin (TOR), belonging to the phosphatidylinositol-3-kinase family, orchestrates energy integration, hormonal responses, and stress signaling to facilitate growth and metabolic processes across eukaryotes [33,34]. Structurally, TOR comprises five conserved domains: the HEAT repeat domain, FAT domain, FRB domain, Ser/Thr kinase domain, and FATC domain [35,36,37,38]. TOR forms two distinct protein complexes, known as TOR complexes 1 (TORC1) and TOR complexes 2 (TORC2), in yeast and mammals [39,40]. While evidence for plant TORC2 is lacking, plants seemingly possess the core components of TORC1, namely RAPTOR and LST8 [34,41,42]. In *Arabidopsis*, RAPTOR1B (TOR 1B) modulates hormone levels during seed germination [43]. TORC1 attenuates ABA signaling while partly activating its downstream pathway. Additionally, ABA inhibits TORC1 activity by phosphorylating RAPTOR at Ser897 [44].

The *SCYL* (*SCY1*-like) gene family comprises evolutionarily conserved proteins characterized by an N-terminal kinase domain, HEAT repeats, and a C-terminal segment. The kinase domains within SCYL proteins are presumed to be inactive, potentially serving regulatory roles in kinase activity or signaling pathways [45,46]. SCYL2, initially identified in animals, plays a crucial role in *Xenopus tropicalis* development [47,48], and is essential for neuronal function in the murine brain; its deletion results in the degeneration of multiple neuronal populations [46,49]. In *Arabidopsis*, both *SCYL2A* and *SCYL2B* redundantly regulate plant growth, with mutations in both resulting in severe dwarfism [50]. *OsSCYL2* in rice participates in the regulation of plant innate immunity as a conserved component of lattice-protein encapsulated vesicles [51]. Additionally, Ppk32, a pseudo-kinase within the SCYL family, modulates the TOR signaling pathway in fission yeast [52].

Seed germination significantly influences rice yield and quality, underscoring the importance of investigating seed germination-associated genes and elucidating their mechanisms. While previous research has elucidated numerous genes involved in the germination pathway, limited attention has been given to the role of *OsSCYL2* in rice. This study aims to fill this gap by identifying a novel protein that enhances seed germination. Furthermore, our findings demonstrate the interaction between OsSCYL2 and OsTOR, showcasing their collective role in enhancing rice seed germination by modulating ABA hormone homeostasis. These insights not only contribute to a deeper understanding of gene functions but also lay the groundwork for developing improved rice varieties with superior germination characteristics.

## 2. Results

### 2.1. Bioinformatics Analysis of OsSCYL2

**Figure 1:**
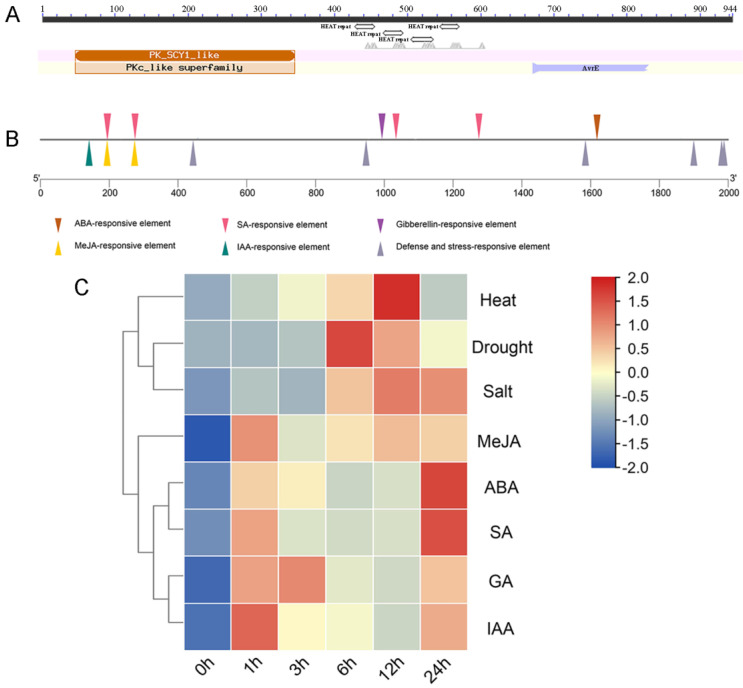
Bioinformatics analysis of OsSCYL2. (**A**) protein structure analysis of OsSCYL2. (**B**) The analysis of cis-elements in the promoter regions of *OsSCYL2*. (**C**) Heatmap of *OsSCYL2* expression in different treatments.

Analysis of the conserved domains of the OsSCYL2 protein reveals that it belongs to the SCYL protein family, with an N-terminal kinase domain, HEAT repeats, and a C-terminal fragment (Figure 1A). The analysis of cis-elements in the promoter regions of *OsSCYL2* was conducted using the PlantCARE online website and the results showed the presence of multiple cis-elements (Figure 1B), including MeJA, auxin, salicylic acid, gibberellin, abscisic acid, and stress-related responsive elements. To further elucidate the responsiveness of *OsSCYL2* to abiotic stresses and phytohormones, this study examined the expression profile of the *OsSCYL2* gene under three abiotic stress conditions (salt, drought, and heat) and exposure to various phytohormones (MeJA, ABA, SA, GA, and IAA). As depicted in Figure 1C, *OsSCYL2* exhibited significant upregulation in response to both abiotic stresses and hormonal treatments. Specifically, the transcriptional abundance of *OsSCYL2* peaked at 12 h following heat and salt treatment, while the maximal induction occurred at 6 h following drought treatment. Furthermore, exposure to different hormones led to a significant increase in *OsSCYL2* expression as early as 1 h post-treatment, with distinct temporal dynamics observed for each hormone. These findings indicated a positive regulatory role of *OsSCYL2* in orchestrating growth and defense mechanisms.

### 2.2. OsSCYL2 Affects the Speed of Seed Germination

**Figure 2:**
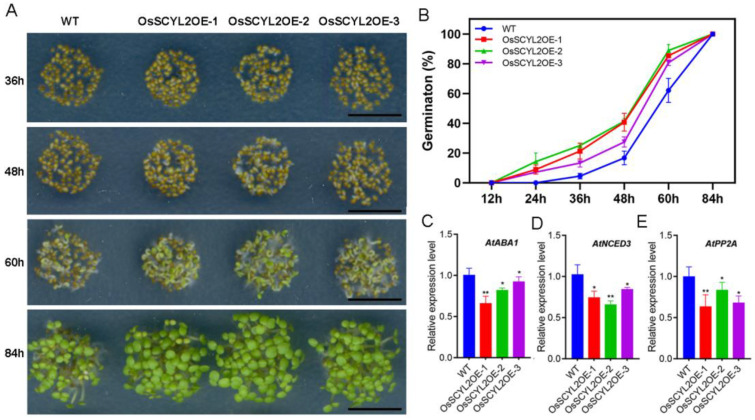
*OsSCYL2* positively regulates seed germination in *Arabidopsis*. (**A**) The phenotype of WT and transgenic *Arabidopsis* (OsSCYL2OE-1, -2, and -3) at 36, 48, 60, and 84 h after sown. Scale bars: 0.5 cm. (**B**) Seeds germination rate of *Arabidopsis* seeds (WT, OsSCYL2OE-1, -2, and -3). Seeds were sterilized and germinated on 1/2 MS medium and germinated seeds were counted after 12 h. Values are expressed as mean ± SD of three biological replicates (n = 50). The expression levels of *AtABA1* (**C**), *AtNED3* (**D**), and *AtPP2A* (**E**) in *Arabidopsis* seeds (WT, OsSCYL2OE-1, -2, and -3) germinated for 48 h. Values are presented as means ± SD (n = 3). (Student’s *t*-test; * *p* < 0.05, ** *p* < 0.01).

In a prior study, three homozygous transgenic lines overexpressing *OsSCYL2* were characterized [53], serving as the experimental material for subsequent investigations. Seed germination assays were conducted to assess the functional role of *OsSCYL2*. The findings revealed a significantly accelerated germination rate in OsSCYL2OE-1, -2, and -3 compared to the wild-type (WT) (Figure 2A,B). Specifically, transgenic *Arabidopsis* seeds (OsSCYL2OE-1, -2, and -3) commenced germination at 12 h, whereas WT initiated germination at 24 h. By the 48 h mark, the germination rates of OsSCYL2OE-1, -2, and -3 reached 21.30%, 25.05%, and 27.43%, respectively, surpassing the WT rate of 16.72%. At 60 h, the germination rates further increased to 85.42%, 88.98%, and 80.7% for OsSCYL2OE-1, -2, and -3, respectively, while WT seeds reached 62.13%. Complete germination was achieved by all seeds within 84 h. Concurrently, quantitative real-time PCR (qRT-PCR) analyses indicated significantly reduced expression levels of ABA biosynthesis-related genes (*ABA1*, *NCED3*, and *PP2A*) in transgenic *Arabidopsis* seeds after 48 h of germination (Figure 2C–E). These findings suggested that *OsSCYL2* overexpression might attenuate the expression of ABA-related genes, thereby enhancing the germination kinetics of *Arabidopsis* seeds.

**Figure 3:**
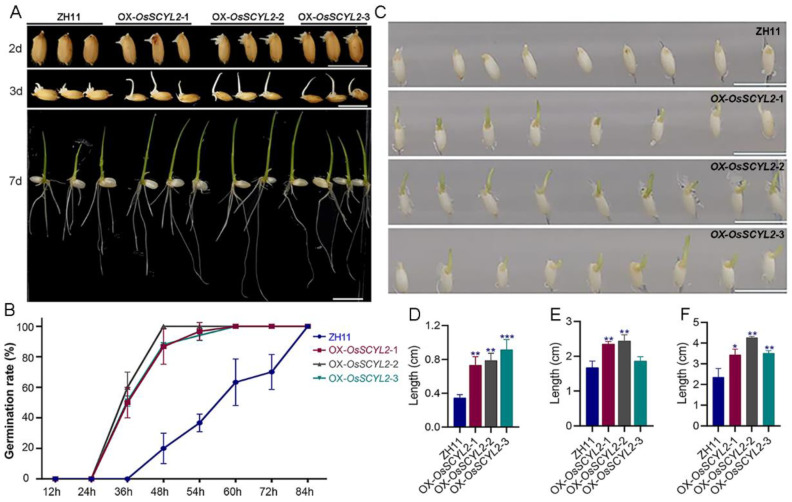
*OsSCYL2* positively regulates seed germination in rice. (**A**) The phenotype of ZH11 and overexpression lines (OX-OsSCYL2-1, -2, and -3) at 2, 3, and 7 days after imbibition. Scale bars: 1 cm. (**B**) Seeds germination rate of ZH11 and overexpression lines (OX-OsSCYL2-1, -2, and -3). The grains were soaked in distilled water and the germination grains were counted after 12 h of imbibition. (**C**) Phenotype of ZH11 and overexpression lines (OX-OsSCYL2-1, -2, -3) at 1/2 MS medium. Scale bars: 1 cm. (**D**) The sprout length of ZH11 and overexpression lines at 3 days after imbibition. The plant height (**E**) and the longest root length (**F**) of ZH11 and overexpression lines at 7 days. Values are presented as means ± SD (n = 3) (Student’s *t*-test; * *p* < 0.05, ** *p* < 0.01,*** *p* < 0.001).

Furthermore, the germination experiments were conducted on rice seeds. Consistently, OX-OsSCYL2-1, -2, and -3 exhibited significantly accelerated germination compared to ZH11 (Figure 3A,B). Rice seeds overexpressing OX-OsSCYL2-1, -2, and -3 initiated germination at 24 h, while ZH11 seeds commenced germination at 36 h. By the 48 h mark, the germination rates of OX-OsSCYL2-1, -2, and -3 had surged to 86.7%, 88.2%, and 100%, respectively, whereas ZH11 seeds only achieved a 20% germination rate. Subsequently, at the 54 h interval, the germination rates of OX-OsSCYL2-1, -2, and -3 reached 96.7%, 94.1%, and 100%, respectively, whereas ZH11 seeds only attained a 36.7% germination rate. Seeds overexpressing *OsSCYL2* reached 100% germination at the 60 h mark, while ZH11 achieved the same rate at 84 h. Concurrently, germination experiments of husk-free seeds were carried out in sterile conditions. Seeds overexpressing *OsSCYL2* exhibited accelerated germination in contrast to ZH11 (Figure 3C), aligning with the germination kinetics observed in husked seeds (Figure 3A). Substantial differences in plant stature were observed between ZH11 and transgenic seedlings. Specifically, at 3 days post-germination, the sprout heights of OX-OsSCYL2-1, -2, and -3 measured 0.73, 0.79, and 0.91 cm, respectively, surpassing that of ZH11 at 0.344 cm (Figure 3D). By day 7 post-germination, the plant heights of OX-OsSCYL2-1, -2, and -3 reached 2.35, 2.43, and 1.87 cm, respectively, significantly exceeding ZH11’s height of 1.67 cm (Figure 3E). Moreover, the longest root lengths of OX-OsSCYL2-1, -2, and -3 were 3.43, 4.25, and 3.52 cm, respectively, markedly surpassing ZH11’s length of 2.36 cm (Figure 3F).

### 2.3. Overexpression of OsSCYL2 Resulted in Changes in ABA-Regulated Genes

**Figure 4:**
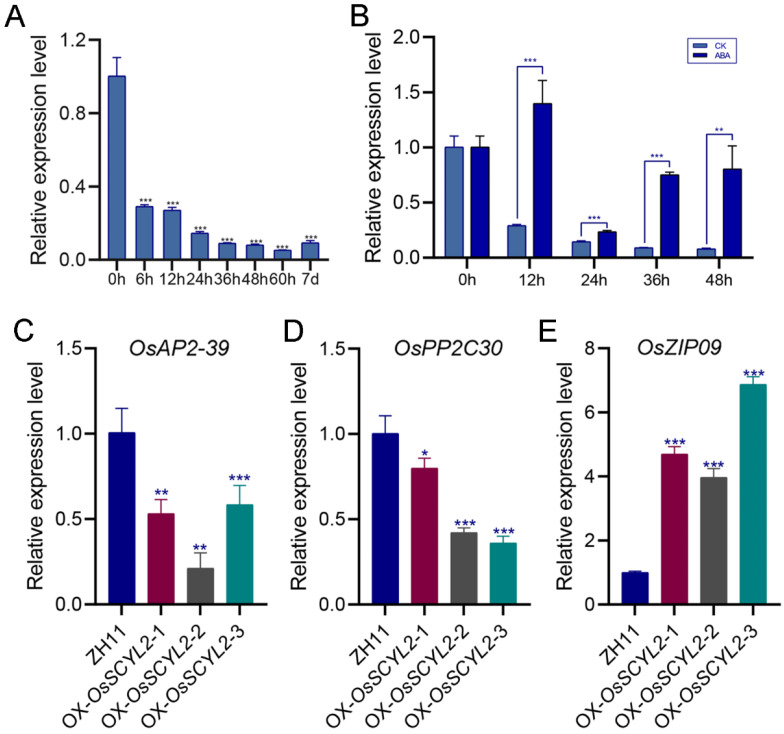
Expression pattern analysis of *OsSCYL2*. (**A**) The expression levels of *OsSCYL2* during seed germination. (**B**) The expression levels of *OsSCYL2* after 10 μM ABA treatment. The expression levels of *OsAp2-39* (**C**), *OsPP2C30* (**D**), and *OsbZIP09* (**E**) in ZH11 and overexpression lines (OX-OsSCYL2-1, -2, and -3). Values are presented as means ± SD (n = 3) (Student’s *t*-test; * *p* < 0.05, ** *p* < 0.01, *** *p* < 0.001).

The expression pattern of *OsSCYL2* during seed germination was detected by qRT-PCR, revealing continuous expression throughout this process (Figure 4A). However, as germination progressed, *OsSCYL2* expression gradually declined, reaching a low level after 36 h. ABA, a key hormone in seed germination regulation, was found to influence *OsSCYL2* expression significantly, as evidenced by a notable increase in *OsSCYL2* expression in germinated seeds treated with 10 μM ABA (Figure 4B). These findings suggested a correlation between *OsSCYL2* expression and ABA levels during seed germination, with exogenous ABA application capable of inducing *OsSCYL2* expression.

Real-time quantitative studies were conducted to investigate changes in ABA-regulated genes in transgenic materials. It was observed that, compared to ZH11, the expression levels of the ABA-related genes *OsAP2-39* [54] and *OsPP2C*-*30* [55], which are known to negatively regulate seed germination, were lower in OX-OsSCYL2-1, -2, and -3 (Figure 4C,D), whereas the expression of *OsZIP09*, a positive regulator of seed germination [56], was higher (Figure 4E). These findings suggested that *OsSCYL2* might modulate the expression of ABA pathway-related genes, thereby influencing ABA content and ultimately affecting seed germination.

### 2.4. OsSCYL2 Regulates MAPK and Plant Hormones Signaling Pathways

**Figure 5:**
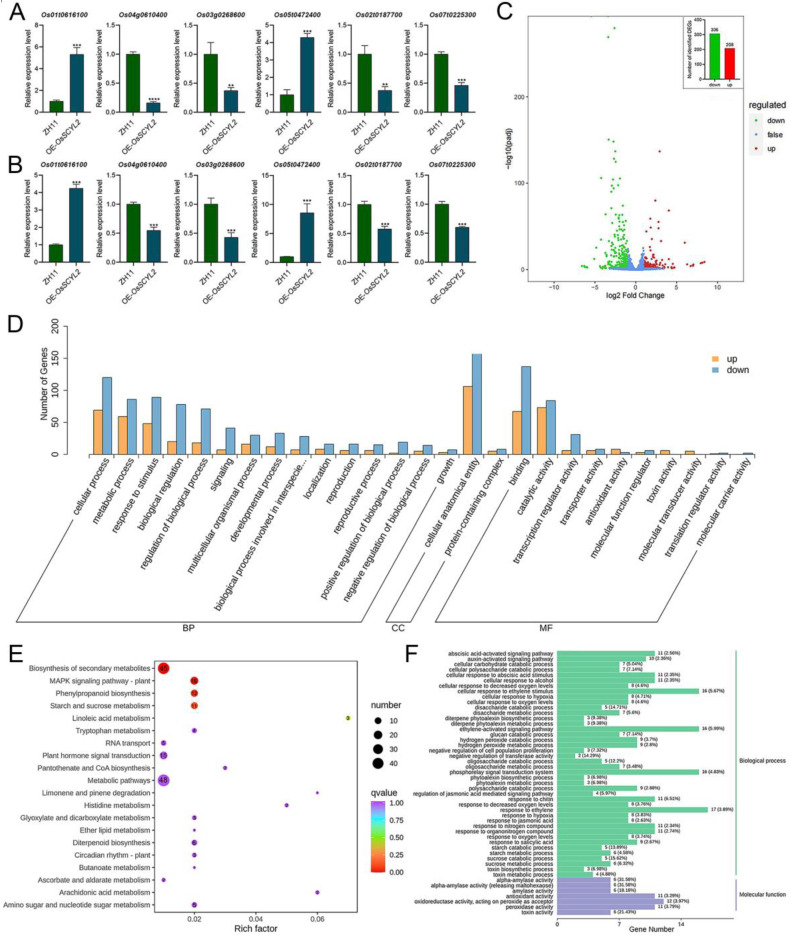
The involvement of differentially expressed genes (DEGs) in plant hormone signal transduction and MAPK signal pathways. The expression profiles of six genes are depicted using RNA-seq (**A**) and validated through qRT-PCR (**B**) (Student’s *t*-test; ** *p* < 0.01, *** *p* < 0.001, **** *p* < 0.0001). A volcano plot (**C**) displays the DEGs between overexpression lines and ZH11. (**D**) GO classification categorizes the DEGs into biological processes (BP), cellular components (CC), and molecular functions (MF). The KEGG (**E**) and GO (**F**) enrichment analysis. The size and color of the dots reflect the number and significance of DEGs, respectively.

RNA-seq analysis, commonly known as transcriptomics, offers a valuable tool for investigating the intricate molecular regulatory mechanisms governing gene expression. Transcriptomic datasets from both ZH11 and *OsSCYL2* overexpression lines were curated to explore differentially expressed genes (DEGs). To validate the accuracy and reproducibility of our RNA-seq findings, qRT-PCR analysis was conducted on six selected genes. It was observed that there existed a high degree of concordance between the relative changes observed in gene expression and those identified through sequencing (Figure 5A,B), thereby affirming the reliability of our dataset. This validation underscores the utility of transcriptomic data for DEG comparison. The comprehensive distribution of DEGs is depicted in the volcano plot (Figure 5C), revealing a total of 514 DEGs, comprising 306 downregulated genes and 208 upregulated genes. Among these, 284 upregulated genes and 173 downregulated genes were enriched in cellular component categories, while 295 upregulated genes and 675 downregulated genes were associated with bioprocesses, and 452 upregulated genes and 274 downregulated genes were implicated in molecular function (Figure 5D). The substantial alterations observed in numerous genes suggested that variations in *OsSCYL2* expression could exert broad regulatory effects on plant germination.

KEGG and GO analyses facilitate the exploration of fundamental biological processes and regulatory mechanisms within gene expression datasets. Notably, KEGG analysis revealed the involvement of DEGs in diverse pathways, including the biosynthesis of secondary metabolites (involving 45 genes), metabolic pathways (involving 48 genes), MAPK signaling pathway (involving 16 genes), and plant hormone signal transduction (involving 15 genes) (Figure 5E). Concurrently, gene ontology analysis highlighted the enrichment of DEGs in various processes such as the phosphorely signal transduction system, cellular response to abscisic stimulus, oxidoreductase activity (acting on peroxide as acceptor), and antioxidant activity (Figure 5F). Notably, among these DEGs, 15 genes were implicated in the ABA-activated signaling pathway (Table 1). For instance, Os03g0268600-01 and Os04g0610400-01 have been identified as positive regulators of ABA signaling, exerting a negative influence on seed germination. Their expression in transgenic rice is notably downregulated, suggesting a potential role for *OsSCYL2* in modulating rice germination through participation in the ABA pathway.

### 2.5. OsSCYL2 Interacted with OsTOR in Rice

**Figure 6:**
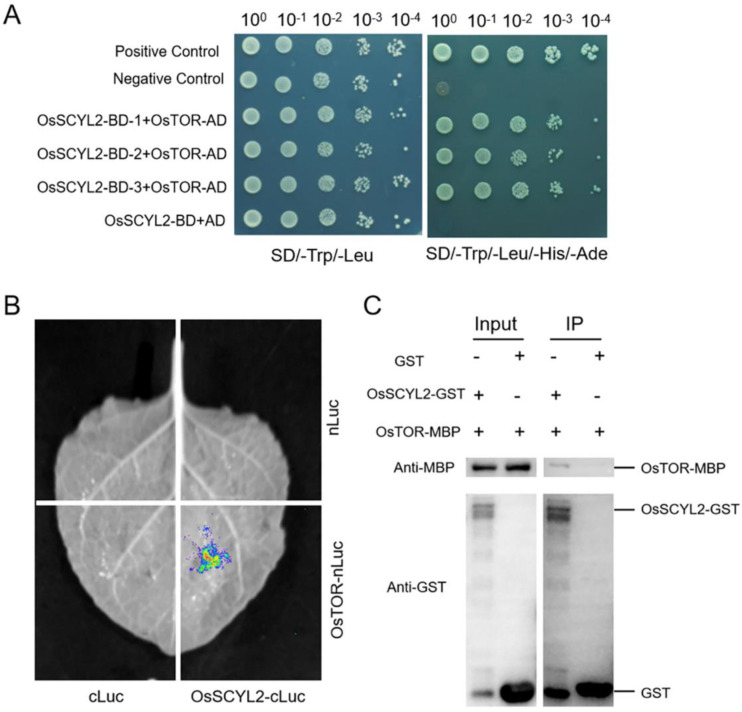
Interaction of OsSCYL2 with OsTOR. (**A**) The interaction between OsSCYL2 and OsTOR was validated through the yeast two-hybrid method, with normal growth observed on SD-Trp/Leu/His/Ade plates indicating their interaction. (**B**) The interaction between OsSCYL2 and OsTOR was confirmed via split-luciferase complementation assay, with fluorescence indicating their interaction. (**C**) Direct binding of OsSCYL2 with OsTOR was verified through GST pull-down assay. The recombinant TOR-MBP protein was pulled down by GST-OsSCYL2, but not GST.

To explore the interaction partners of OsSCYL2, we employed a combination of experimental techniques, including yeast two-hybrid, split-luciferase, and GST pull-down assays. In the yeast two-hybrid assay results, all three co-transformed yeast strains (carrying OsSCYL2-BD and OsTOR-AD constructs) exhibited normal growth on the SD/-Ade/-His/-Leu/-Trp medium, indicating their interaction within the yeast cells (Figure 6A). Furthermore, we conducted the split-luciferase assay using a transiently expressed *N. benthamiana* system. Among the various combinations tested, only OsSCYL2-cLUC and OsTOR-nLUC produced characteristic fluorescence, while other combinations showed no luciferase signals. This observation confirms the interaction between OsSCYL2 and OsTOR in split-luciferase assays (Figure 6B). To validate the direct interaction between OsSCYL2 and OsTOR, a pull-down assay was performed. Notably, Os-TOR-MBP was specifically pulled down by OsSCYL2-GST, indicating an interaction between TOR and OsSCYL2 in vitro, as opposed to GST alone, which did not show such binding (Figure 6C). In summary, our comprehensive analysis demonstrates the interaction of OsSCYL2 with TOR.

## 3. Discussion

Previous research has established that *OsSCYL2* is a member of the SCYL-like gene family, characterized by its evolutionarily conserved nature and potential involvement in regulating kinase activity or signaling pathways [45,46]. In our investigation, we observed a significant acceleration in the germination rate of OsSCYL2OE-1, -2, and -3 compared to the wild-type, as illustrated in Figure 2. Specifically, rice seeds from OX-OsSCYL2 lines exhibited germination 12 h earlier than those from the ZH11 line, with full germination rates of both OX-OsSCYL2 lines and ZH11 reaching 60 h and 84 h, respectively (Figure 3). Additionally, in *Arabidopsis*, AtSCYL2 has been identified as a regulator of plant growth and development [50], while in rice, OsSCYL2 plays a role in modulating plant innate immunity as a conserved lattice-protein encapsulated vesicle component [51]. These findings collectively suggest that overexpression of OsSCYL2 facilitates plant seed germination, thereby contributing to the expanding understanding of the functional diversity within the *SCYL* gene family.

The comprehensive exploration of cis-elements within the promoter regions of the *OsSCYL2* gene sheds light on the intricate regulatory mechanisms governing its expression. The identification of multiple cis-elements, including MeJA, auxin, salicylic acid, gibberellin, abscisic acid, and other stress-related responsive elements, underscores the multifaceted nature of *OsSCYL2* regulation within the plant system (Figure 1D). Furthermore, the significant upregulation of *OsSCYL2* gene expression levels under various stress conditions (salt, drought, and heat) and exposure to different hormonal stimuli (MeJA, ABA, SA, GA, and IAA) as depicted in Figure 1E, highlights the pivotal role of *OsSCYL2* in orchestrating plant growth and stress responses. This dynamic modulation of *OsSCYL2* expression in response to diverse environmental cues underscores its versatility as a molecular player in mediating plant adaptive responses. Moreover, the elucidation of OsSCYL2 expression dynamics during seed germination reveals a nuanced interplay with ABA signaling. The observed decrease in *OsSCYL2* expression concomitant with declining ABA content during germination, coupled with the ability of exogenously applied ABA to induce *OsSCYL2* expression (Figure 4), underscores the intricate regulatory interplay between *OsSCYL2* and ABA-mediated signaling pathways during early developmental stages. Taken together, these findings underscore the intricate regulatory landscape governing *OsSCYL2* expression, highlighting its responsiveness to a myriad of hormonal and environmental cues, with ABA emerging as a central regulator. The elucidation of *OsSCYL2’s* regulatory network provides valuable insights into its functional role in plant growth, stress responses, and developmental processes.

The intricate regulatory network governing seed germination involves a plethora of TFs, among which ABA-responsive elements play a crucial role. Studies have elucidated the involvement of various TFs, including ABI3, AP2, bZIPs, and WRKY TFs, in the ABA-mediated regulation of germination, highlighting their significance in coordinating this essential biological process [4,27,30,57]. In this study, the focus shifts to *OsSCYL2*, a novel player in seed germination regulation, demonstrating its pivotal role in modulating the ABA pathway to promote germination. Transcriptome analysis following the overexpression of *OsSCYL2* revealed substantial alterations in key pathways, including secondary metabolite pathways, metabolic pathways, MAPK signaling, and phytohormone signal transduction pathways within rice (Figure 5). Notably, 15 genes associated with the ABA pathway exhibited significant changes upon *OsSCYL2* overexpression, exemplified by *OsAP2*-*39*, *OsPP2C*-*30*, and *OsbZIP09* (Table 1). The identified genes, known for their roles in promoting seed germination, further validate OsSCYL2’s impact on ABA signaling modulation. For instance, ABI3, a positive regulator of ABA signaling, has been implicated in conferring sensitivity to ABA during seed germination, as evidenced by studies on abi3 mutants [19,20]. Conversely, *OsAP2*-*39*, a TF harboring the AP2 domain, exerts a negative regulatory influence on seed germination [21], while *OsbZIP09*, a member of the bZIP gene family, is known to promote germination [23,24,25,29]. The qRT-PCR analysis provided additional insights into the regulatory dynamics, revealing differential expression patterns of key ABA-related genes in OsSCYL2-overexpressing rice lines compared to the control. The downregulation of *OsAP2*-39 and *OsPP2C*-30, coupled with the upregulation of *OsbZIP09* in OsSCYL2-overexpressing lines, further corroborates OsSCYL2’s role as a negative regulator of ABA signaling, thereby influencing seed germination outcomes. Collectively, these findings underscore the intricate regulatory interplay orchestrated by *OsSCYL2* in seed germination, particularly through its modulation of the ABA pathway.

The TOR pathway stands as a pivotal regulator across eukaryotic organisms, orchestrating a myriad of growth and metabolism-related processes. As underscored by prior research [33,58], TOR’s influence extends ubiquitously, exerting its regulatory control over essential cellular functions. In the context of our investigation, we delved into the interplay between TOR and OsSCYL2, shedding light on their potential interaction and its implications for plant biology. Our protein–protein interaction assays provided compelling evidence of the physical association between OsSCYL2 and TOR. Co-transformation experiments, utilizing yeast strains expressing OsSCYL2 and TOR constructs, demonstrated normal growth on selective media, indicating their compatibility and potential interaction (Figure 6A). Furthermore, the generation of fluorescence signals upon the co-expression of OsSCYL2-cLUC and OsTOR-nLUC underscored the in vivo interaction between these proteins, complementing the findings from in vitro assays (Figure 6B). Notably, the biochemical assay revealed the direct binding of OsTOR to OsSCYL2, as evidenced by the pull-down of OsTOR-MBP by OsSCYL2-GST (Figure 6C). These comprehensive findings unequivocally establish the interaction between OsSCYL2 and TOR, both in vitro and in vivo, thus implicating OsSCYL2 as a novel player in TOR signaling in plants. The identification of Ppk32, a homolog of SCYL1/2, as a regulator of TOR signaling in fission yeast serves as a notable precedent, highlighting the evolutionary conservation of TOR regulatory mechanisms across species [52]. Furthermore, the involvement of TOR1B in modulating hormone levels during seed germination in *Arabidopsis* underscores the significance of TOR signaling in plant developmental processes, particularly in germination regulation [43]. Building upon these insights, our study advances the understanding of SCYL2’s regulatory role within the TOR pathway in plants. By elucidating its involvement in modulating hormone levels during seed germination and impacting plant growth and development, our findings provide a foundation for further exploration into the intricate molecular mechanisms governing TOR signaling in plants. Moreover, they offer potential avenues for targeted manipulation of TOR signaling to optimize plant growth, development, and stress responses, thereby offering promising prospects for agricultural enhancement and crop improvement strategies.

## 4. Materials and Methods

### 4.1. Plant Materials and Growth Conditions

The *Arabidopsis* Columbia-0 and Zhonghua 11 (ZH11, *O. sativa* ssp. Japonica) were employed as wild-type and the recipients for genetic transformation in the present study. The *OsSCYL2*-pCAMBIA1301 vector was constructed by integrating the full-length coding sequence (CDS) of the cloned *OsSCYL2* into the pCAMBIA1301 vector, which was subsequently introduced into rice callus and *Arabidopsis* through *Agrobacterium*-mediated transformation [53,59]. All primers used for qRT-PCR and vector construction are presented in Table S1. Overexpression of *OsSCYL2* lines was identified by qRT-PCR with specific primers (Table S1) at the RNA levels, and histochemical staining of GUS in transgenic rice at the protein levels [60,61,62]. Three homozygous seed lines overexpressing *OsSCYL2* were identified and used for subsequent studies. *Arabidopsis* seedlings were cultivated at 22 °C with a 16 h light/8 h dark cycle, while rice plants were cultivated in the paddy field at Anhui Agricultural University in Hefei in the summer and in a greenhouse (16 h light/8 h dark, 70–80% humidity, 28 °C) in the winter.

### 4.2. Bioinformatics and Expression Analysis of OsSCYL2

The structural domain of the OsSCYL2 protein was predicted by NCBI Conserved domains (https://www.ncbi.nlm.nih.gov/Structure/cdd/, accessed on 12 January 2022). To predict putative cis-regulatory elements in the promoter regions of *OsSCYL2*, the 2000 bp upstream of the start codon sequences of *OsSCYL2* was obtained from the rice genomic sequence, and the promoter sequence was analyzed via the PlantCARE online website (https://bioinformatics.psb.ugent.be/webtools/plantcare/html/, accessed on 24 January 2022). To explore the expression level of the *OsSCYL2* in different stresses and hormones, the three-leaf stage rice seedlings were selected and treated with high-temperature treatment (42 °C), drought, salt (150 mM NaCl), and different hormones (50 μM MeJA, 100 μM ABA, 100 μM SA, 100 μM GA, and 50 μM IAA), the leaves were harvested at different times (0, 1, 2, 4, 8, and 24 h). The RNA of each sample was extracted as described above and qRT-PCR analyzed the changes in the expression level of *OsSCYL2*.

### 4.3. Seed Germination Assay and ABA Treatment Assay

To assess the rate of seed germination, the sterilized *Arabidopsis* seeds were placed on 1/2 MS medium plates. Germinated seeds were counted after 12 h of imbibition, while the phenotypes were observed and recorded. The sterilized rice seeds were individually sown on Petri dishes containing distilled water. The rice seeds were cultivated in the greenhouse, and germination grains were counted after 12 h imbibition. Germination photographs were captured at 2-, 3-, and 7-day intervals and the measurements were taken to determine the length of shoots, seedlings, and roots. For the ABA treatment assay, seeds were harvested at 0, 6, 12, 24, 36, 48, and 60 h after imbibition. Additionally, another set of seeds were collected at 0, 6, 12, 24, 36, and 48 h following imbibition with 10 μM ABA solution. Furthermore, 7-day-old seedlings were harvested, and an additional set of these seedlings was collected at 0, 3, 6, and 12 h after treatment with a 10 μM ABA solution. Three experiments were replicated for each sample, and each group of samples was frozen in liquid nitrogen and stored at −80 °C.

### 4.4. RNA Extractions, RNA Library Construction, and Sequencing

Total RNA was extracted from the samples using Trizol reagent (Sangon Biotech, Shanghai, China), and the transcript levels of each gene were assessed by qRT-PCR with specific primers (Table S1), utilizing *OsActin1* as the internal control. For RNA library construction, the mRNAs were isolated separately using oligo (dT) ligated beads and fragmented into small pieces. Subsequently, first- and second-strand cDNA synthesis was performed with random hexamer primers, reverse transcriptase, RNase H, and DNA polymerase I. The double-stranded cDNA was purified using the AMPure XP system and subjected to end repair by ligation with paired-end adaptors. The final cDNA library underwent PCR enrichment, and library quality was assessed using the Agilent Bioanalyzer 2100 system (Agilent, Santa Clara, CA, USA) before sequencing on an Illumina HiSeq platform (Agilent, Santa Clara, CA, USA). Each sample underwent three experimental replicates and three biological replicates.

### 4.5. KEGG and GO Analyses

The expression levels of each transcript were normalized to Reads Per Kilobase per Million reads (RPKM) [63], using a criterion of |log_2_ (fold-change)| ≥ 1 and *p* value < 0.05 to identify differentially expressed genes (DEGs). The KEGG (http://www.genome.jp/kegg/, accessed on 12 December 2022) and GO databases (http://www.geneontology.org/, accessed on 12 December 2022) were utilized for pathway and functional annotations of DEGs.

### 4.6. Yeast Two-Hybrid Assay

To verify the interaction between OsSCYL2 and OsTOR in yeast, the OsSCYL2 was fused with the pGBKT7 (BD) vector, while OsTOR was fused with the pGADT7 (AD) vector before being co-transformed into the yeast strain Y2H Gold. Yeast transformants were cultured on SD/-Leu/-Trp medium, followed by serial dilution onto SD/-Leu/-Trp and SD/-Ade/-His/-Leu/-Trp media plates for 3–5 days at 30 °C. Bait toxicity and recombinant plasmid activation tests were conducted on medium plates (SD/-Leu, SD/-Trp) at 30 °C. Co-transformed yeast strain Y2H Gold (AD-T and BD-53) served as the positive control, while Y2H Gold (AD-T and BD-Lam) acted as the negative control.

### 4.7. Split-Luciferase Complementation Assay (Split-LUC)

The split-luciferase complementation assay, widely utilized for comprehending physical contacts among signaling proteins, effectively detects protein interactions in plant cells [64]. OsSCYL2 and OsTOR coding sequences were fused to luciferase C-terminal fragment (OsSCYL2-cLUC) and N-terminal fragment (OsTOR-nLUC), respectively. Subsequently, the recombinant plasmid was introduced into *Agrobacterium*, employing the transient expression method in *N. benthamiana* as detailed by Li et al. [65]. Following the application of luciferin (100 mM) to the backside of *N. benthamiana* leaves and a 10 min incubation in darkness, the leaves were observed and photographed using a CCD (low-light cooled charge-coupled device) imaging apparatus NightSHADE LB 985 (BERTHOLD, Bad Wildbad, Germany).

### 4.8. Glutathione Stransferase (GST) Pull-Down Assay

A GST pull-down assay confirmed the interaction between OsSCYL2 and OsTOR. They were, respectively, fused with pGEX-4T-1 and pMAL-c4X vectors, and transformed into *Escherichia coli* BL21. After overnight incubation at 16 °C to express the recombinant protein, sodium dodecyl sulfate-polyacrylamide gel electrophoresis (SDS-PAGE) detected the protein. Equal quantities of OsSCYL2-GST proteins or GST proteins, along with 100 μL of GST sefinose^TM^ resin (Sangon Biotech, Shanghai, China), were incubated in 500 μL GST binding buffer for 2 h at 4 °C. Subsequently, OsTOR-MBP proteins were added to the mixture and incubated overnight at 4 °C. Enhanced chemiluminescence chemical exposure analysis was performed using an anti-MBP antibody and an anti-GST antibody after extensive washing and boiling for the detection of target proteins.

## 5. Conclusions

In this study, seed germination, a complex process influenced by various factors including transcription factors and specific proteins, was investigated. Our findings revealed that *OsSCYL2* acts as a positive regulator of rice seed germination. Additionally, exogenous application of ABA during both seed germination and post-germination growth demonstrated its ability to induce *OsSCYL2* expression. Notably, OX-OsSCYL2 lines exhibited a significantly accelerated germination rate compared to the ZH11 control line. Analysis of OX-OsSCYL2 seedlings revealed significant alterations in 15 ABA pathway-related genes, resulting in corresponding changes in metabolic pathways, MAPK signaling, and phytohormone signal transduction pathways. Furthermore, both in vivo and in vitro experiments confirmed the interaction between OsSCYL2 and OsTOR, suggesting a role for *OsSCYL2* in negatively regulating ABA response genes, influencing rice germination speed, and contributing to seed germination processes.

## Figures and Tables

**Table 1:** Identification of ABA-related DEG.

ID	Description	KEGG	*p* Value	Regulated
*Os01g0511000-01*	Predicted: LOB domain-containing protein 29	K13945	2.87563 × 10^−48^	down
*Os04g0208200-01*	Predicted: senescence-specific cysteine protease SAG39	K16292	0.001279091	up
*Os05g0545400-01*	Mitogen-activated protein kinase kinase kinase 19	K20716	2.04441 × 10^−28^	down
*Os02g0187700-00*	Myb-like DNA-binding domain containing protein	K09422	9.00346 × 10^−19^	down
*Os06g0637500-02*	Predicted: transcription factor MYB	K09422	1.52605 × 10^−18^	down
*Os01g0699600-01*	Mitogen-activated protein kinase kinase kinase	K20716	1.02578 × 10^−16^	down
*Os04g0586500-01*	Predicted: receptor-like protein kinase FERONIA	--	7.77913 × 10^−12^	down
*Os03g0268600-01*	Protein phosphatase 2C 30	K14497	2.91137 × 10^−7^	down
*Os05g0109825-00*	Predicted: E3 ubiquitin-protein ligase RING1	K11982	2.08451 × 10^−5^	down
*Os09g0243200-01*	Predicted: E3 ubiquitin-protein ligase CIP8	K22378	0.00023672	up
*Os05g0545300-01*	Predicted: mitogen-activated protein kinase kinase kinase 17/18	K20716	0.001312045	down
*Os05g0494600-01*	Predicted: EID1-like F-box protein 3	--	0.002511623	down
*Os11g0521900-01*	Predicted: RNA polymerase II C-terminal domain phosphatase-like 3/4	K18999	0.002660793	down
*Os04g0610400-01*	APETALA-2-Like transcription factor gene; *OsAP2-39*	K09286	1.9587 × 10^−132^	down
*Os05g0472400-01*	Zinc transporter 9 isoform X1	K14709	9.95893 × 10^−18^	up
*Os01g0963600-01*	Abscisic acid-stress-ripening-inducible 6 protei	--	3.81499 × 10^−50^	down
*Os02g0657000-01*	AP2/EREBP-type transcription factor; ABA-responsive *DREB* gene	K09286	5.08219 × 10^−12^	down

## Data Availability

Data is contained within the article and Supplementary Materials.

## Supplementary Materials

The following supporting information can be downloaded at: https://www.mdpi.com/article/10.3390/plants13081088/s1. Table S1. All primers were used for qRT-PCR and vector construction.
