# Supplementary material for: OsSCYL2 is Involved in Regulating ABA Signaling-Mediated Seed Germination in Rice
Source: Plants (Basel). 2024 Apr 12;13(8):1088. doi: 10.3390/plants13081088 (PMC11054224; doi:10.3390/plants13081088)
Supplement: Supplementary file 1 [file plants-13-01088-s001.zip › plants-2920495-supplementary.pdf]

Supplementary Materials

OsSCYL2 Is Involved in Regulating ABA Signaling-Mediated Seed Germination in Rice

Minyan Xu †, Wei Zhang †, Yuhuan Jiao, Qing Yang, Meng Chen, Hu Cheng, Beijiu Cheng and Xin Zhang *

National Engineering Laboratory of Crop Stress Resistance Breeding, School of Life Sciences, Anhui Agricultural University, Hefei 230036, China; xu285083469@163.com (M.X.); antoniatan@sina.com (W.Z.); m18355678848@163.com (Y.J.); y1162999081@163.com (Q.Y.); chenmengcm2022@163.com (M.C.); 15212917461@163.com (H.C.); cbj@ahau.edu.cn (B.C.)

* Correspondence: xinzhang@ahau.edu.cn

† These authors contributed equally to this work.

Table S1. All primers were used for qRT-PCR and vector construction.

Primer name	Sequence (5' to 3')
OsActin1-qF	TCTCTCTGTATGCCAGTGGTCGT
OsActin1-qR	TCATAGTCCAGGGCGATGTAGG
OsSCYL2-qF	TGGTCAGCGAAACCGAACACTAC
OsSCYL2-qR	CAGAAGCAGATGGAGGAGGAGC
OsPP2C30-qF	ACGAGTTCCTCATCCTGG
OsPP2C30-qR	GAGCACCGTCGTTGGGCG
OsAp2-39-qF	CGTGGCGGAGGGCGGAGGA
OsAp2-39-qR	GGCGGGGCGTGGTGGTGGG
OsZIP09-qF	GAAGGGATAGGGCTGGGAG
OsZIP09-qR	TCATTGTAGGCGGAGGAGA
OsMYB44-qF	CGGGTTCGGGCAGATTTT
OsMYB44-qR	AGGGAGCGAGAGGGAGAG
OsNAC3-qF	GTGTTCTACCACGGCAAGC
OsNAC3-qR	ACAGCACCCAGTCATCCAG
OsSCYL2-GUS-F	GAGAACACGGGGGACTCTTGACCAAAATCACCAAACACAC
OsSCYL2-GUS-R	AACTAGAAATTTACCCTCAGATCTACCAGTAAATCTAGAATAGG
OsSCYL2-BD-F	atggccatggaggccgaattcATGGCGCTGAACATGAAGACG
OsSCYL2-BD-R	ctagttatgcggccgctgcagCAGTAAATCTAGAATAGGTGGCTGC
OsTOR-AD-F	gccatggaggccagtgaattcCTAGATGCGATGTTTTCTGCTGG
OsTOR-AD-R	attcatctgcagctcgagctcTGGAGGATACTCATTATAAGGAGAGATAA
OsSCYL2-cLUC-F	tacgcgtcccggggcggtaccATGGCGCTGAACATGAAGACG
OsSCYL2-cLUC-R	tacgcgtcccggggcggtaccATGGCGCTGAACATGAAGACG
OsTOR-nLUC-F	acgggggacgagctcggtacccCTAGATGCGATGTTTTCTGCTGG
OsTOR-nLUC-R	cgagatctggtcgacactagtTGGAGGATACTCATTATAAGGAGAGATAA
OsSCYL2-GST-F	ccgcgtggatccccggaattcATGGCGCTGAACATGAAGACG
OsSCYL2-GST-R	gatgcggccgctcgagtcgacCAGTAAATCTAGAATAGGTGGCTGCT
OsTOR-MBP-F	gagggaaggatttcagaattcCTAGATGCGATGTTTTCTGCTGG
OsTOR-MBP-R	cagtgccaagcttgcctgcagTGGAGGATACTCATTATAAGGAGAGATAA
